# Development and validation of two prognostic nomograms for predicting survival in patients with non-small cell and small cell lung cancer

**DOI:** 10.18632/oncotarget.19791

**Published:** 2017-08-02

**Authors:** Hai-Fan Xiao, Bai-Hua Zhang, Xian-Zhen Liao, Shi-Peng Yan, Song-Lin Zhu, Feng Zhou, Yi-Kai Zhou

**Affiliations:** ^1^ State Key Laboratory of Environment Health (Incubation), Key Laboratory of Environment and Health, Ministry of Education, Key Laboratory of Environment and Health (Wuhan), Ministry of Environmental Protection, School of Public Health, Tongji Medical College, Huazhong University of Science and Technology, Wuhan, Hubei 430030, China; ^2^ The Department of Cancer Prevention, Hunan Cancer Hospital, Changsha 410006, China; ^3^ The Department of Thoracic Surgery, Hunan Cancer Hospital, Changsha 410006, China

**Keywords:** external validation, nomograms, non-small-cell lung cancer, small-cell lung cancer, treatment regimen

## Abstract

**Purpose:**

This study aimed to construct two prognostic nomograms to predict survival in patients with non-small-cell lung cancer (NSCLC) and small-cell lung cancer (SCLC) using a novel set of clinical parameters.

**Patients and Methods:**

Two nomograms were developed, using a retrospective analysis of 5384 NSCLC and 647 SCLC patients seen during a 10-year period at Xiang Ya Affiliated Cancer Hospital (Changsha, China). The patients were randomly divided into training and validation cohorts. Univariate and multivariate analyses were used to identify the prognostic factors needed to establish nomograms for the training cohort. The model was internally validated via bootstrap resampling and externally certified using the validation cohort. Predictive accuracy and discriminatory capability were estimated using concordance index (C-index), calibration curves, and risk group stratification.

**Results:**

The largest contributor to overall survival (OS) prognosis in the NSCLC nomogram was the therapeutic regimen and diagnostic method parameters, and in the SCLC nomogram was the therapeutic regimen and health insurance plan parameters. Calibration curves for the nomogram prediction and the actual observation were in optimal agreement for the 3-year OS and acceptable agreement for the 5-year OS in both training datasets. The C-index was higher for the NSCLC cohort nomogram than for the TNM staging system (0.67 vs. 0.64, P = 0.01) and higher for the SCLC nomogram than for the clinical staging system (limited vs. extensive) (0.60 vs. 0.53, *P* = 0.12).

**Conclusion:**

Treatment regimen parameter made the largest contribution to OS prognosis in both nomograms, and these nomograms might provide clinicians and patients a simple tool that improves their ability to accurately estimate survival based on individual patient parameters rather than using an averaged predefined treatment regimen.

## INTRODUCTION

Cancer is the second most common cause of death globally and lung cancer is the leading cause of cancer deaths worldwide [1]. Lung cancers can be divided into two categories, non-small cell lung cancer (NSCLC) and small cell lung cancer (SCLC), which account for 85% and 15% of cases, respectively [2]. For patients with early-stage NSCLC, radical resection is the most common and potentially curative treatment, whereas patients with late-stage NSCLC receive a combination of adjuvant chemotherapy with complete resection [3]. Adjuvant radiotherapy and chemotherapy are typically recommended for high risk patients with lung cancer and more aggressive malignancies involving lymph nodes or residual cancer [4]. Some studies used stereotactic ablative radiation therapy for early-stage NSCLC treatment [5]. The treatment for SCLC is less effective than the treatment for NSCLC [6]. The prognosis for patients with lung cancer remains poor with an overall 5-year survival rate of approximately 15% [7]. The discriminatory value of prognostic biological markers is insufficient to predict an individual's overall survival (OS) [8], and survival time has unfortunately improved little in recent decades [9].

The survival time of patients with the same cancer stage varies widely [10, 11]. Insights into the causes of this variation could be applied for development improved predictive models and new treatment strategies. Because of the inability to identify diagnostic biomarkers using traditional strategies, alternative strategies for identifying diagnostic parameters are of paramount importance. Efforts have focused on expanding the scope and number of parameters studied, and then using this expanded informational content for stratification of patients into risk groups, based on the association of these parameters with disease trajectories and patient outcomes. Statistical modeling theory states that, as the number of parameters increases, the patient cohort size incrementally reduces until the cohort comprises only a few or one patient. Personalized patient care improves predictive prognostics and enhances clinicians’ capacity to individualize healthcare. This increases therapeutic efficacy and facilitates early treatment, thereby improving outcomes, which highlights the long-term potential of the strategy to increase the overall quality of healthcare while reducing costs. Prognostic models are based on the statistical analysis of multiple parameters such as age, gender histology, number of harvested lymph nodes, metastatic information, serum diagnostics and treatment-related factors [4, 11–13]. Multiparameter analysis with patient stratification has resulted in prognostic models that have improved our ability to predict lung cancer patient survival. To fulfill the full potential of the strategy, additional schemes for stratifying patient cohorts are urgently needed.

A nomogram is a graphical calculator that is based on regression models, and has become a popular tool for building predictive models. By creating an intuitive graph of a statistical predictive model, nomograms are accurate and precise tools for estimating risk by correlating the relationship between parameters and various cancer prognosis parameters such as metastatic probability, OS, and recurrence probability [14]. The development of personalized predictors of cancer patient survival is of vital importance for clinicians and patients, both of whom are involved in making treatment decisions. For several types of cancers, nomograms generate more precise predictions, compared to the traditional tumor-node-metastasis (TNM) staging classification system [4, 15, 16].

Nomograms covering a wide range of parameters have been constructed for NSCLC and SCLC. Demographic and clinical parameters used to construct nomograms include age, gender occupation, health insurance plan, and diagnostic method. However, few nomograms include the therapeutic regimen parameter as a prognostic factor for predicting OS [6, 17–19]. Furthermore, few studies have specifically focused on OS prognostics among lung cancer patients. We therefore conducted a retrospective study to explore the prognostic value of the therapeutic regimen parameter for predicting OS among lung cancer patients., In this paper, we developed two nomograms that incorporated both the standard and nonstandard parameters of therapeutic regimen, health insurance plan, and diagnostic method to determine if these parameters improve the ability of the nomogram to predict OS outcomes among NSCLC and SCLC patients.

## RESULTS

### The screening process and the clinicopathologic characteristics of the patients

In the primary NSCLC database (comprising 5384 patients), patients who had missing information on clinical stage (661 patients), smoking history (354 patients), clinical T stage (117 patients), clinical N stage (19 patients), and diagnostic method (13 patients) were excluded from the NSCLC database based on screening criteria. Finally, 4220 NSCLC patients and 643 SCLC patients were included.

In the NSCLC cohort, 3149 events (i.e., deaths) occurred during a median follow-up time of 6.5 years (range, 4 days–11.5 years). The median survival time was 2.3 years (95% confidence interval [CI], 2.2-2.4 years). In the SCLC group, 523 events (i.e., deaths) were identified and the median follow-up time was 6.3 years (range, 8 days–10.8 years). The median survival time for the SCLC group was 1.7 years (95% CI, 1.5-1.8 years). The demographic and clinicopathologic characteristics of the NSCLC and SCLC cohorts are listed in Table 1.

**Table 1:** Demographics, clinicopathologic characteristics of patients and univariate analysis in training cohort

		NSCLC(N=4220)				SCLC(N=643)			
		Training set(N=2954)			Validation(N=1266)	Training set (N=450)			Validation(N=193)
		Cases (%)	OS(months)Median(95%CI)	*P*	Cases (%)	Cases (%)	OS(months)Median(95%CI)	*P*	Cases (%)
Gender	Male	2365	26.8(25.8-28.3)	0.672	1012	376	19.4(17-22.7)	0.099	42
	Female	589	29.3(25.6-32.4)		254	74	21.0(16-47.0)		151
Age, years	<60	1935	27.0(25.8-28.9)	0.095	814	305	20.0(17.8-25.0)	0.241	132
	60-70	831	24.8(20.4-32.0)		77	121	19.2(16.8-23.0)		51
	>70	188	27.4(25.7-32.0)		375	24	14.5(10.0-26.0)		10
Smoking habit, pack-y	<30	1048	26.9(24.9-29.0)	0.727	633	218	19.4(17.0-25.0)	0.584	113
	30-40	633	26.5(24.0-29.9)		241	126	19.5(16.0-26.0)		47
	>40	516	28.9(26.7-34.6)		392	106	20.0(16.4-24)		33
Occupation	Enterprise or company employee/worker	662	30.5(26.7-36.0)		252	78	16.0(14-20.0)		41
	Farmer	1027	24.3(22.3-26.9)	0.003	432	184	21.9(18-26.0)	0.478	70
	Public sector employee	960	27.2(24.7-30.6)		435	135	20.0(17-25.2)		55
	Freelance or self-employed	157	28.9(25.1-45.3)		76	26	25.5(19-50.0)		16
	Others	148	32.4(25.9-51.7)		71	27	19.0(15-NA)		11
Education	Primary school or below	666	26.9(24.1-31.8)	<0.001	284	106	19.0(15.7-28.0)	0.002	45
	Junior and Senior high school	1682	28.3(26.7-30.6)		715	249	23.0(20.0-26.0)		103
	Undergraduate or over	436	28.5(23.0-36.0)		189	59	17.4(15.9-25.0)		26
Health insurance	New rural cooperative medical scheme	538	28.3(25.8-32.0)		225	105	25.0(22.0-35)		46
	Urban residents basic medical insurance	210	37.7(29.3-49.0)	<0.001	92	36	23.4(14.0-47)	0.002	15
	Urban employees basic medical insurance	1013	32.8(29.4-37.3)		416	142	19.4(16.2-25)		48
	Self pay	707	25.6(21.6-28.1)		323	102	19.0(15.0-25)		46
	Other	486	20.4(18.0-23.6)		210	65	14.2(11.3-18)		38
Clinical T stage	T1	260	37.2(30.5-52.2)	<0.001	91	—	—	—	—
	T2	1510	32.3(29.3-36.3)		653	—	—	—	—
	T3	547	23.1(19.9-26.7)		241	—	—	—	—
	T4	637	20.4(18.3-22.4)		281	—	—	—	—
Clinical N stage	N0	909	51.8(48.2-60.5)	<0.001	361	—	—	—	—
	N1	369	38.6(30.6-47.0)		164	—	—	—	—
	N2	934	23.0(20.7-24.6)		417	—	—	—	—
	N3	742	17.4(15.5-19.2)		324	—	—	—	—
Clinical M stage	M0	2022	36.1(33.1-38.6)	<0.001	852	—	—	—	—
	M1	932	15.6(14.8-17.8)		414	—	—	—	—
Clinical stage	—/ Limited	—	—	—	—	254	21(19-24.8)	0.069	105
	—/ Extensive	—	—	—	—	196	17(15-22.0)		88
Tumor location	Right upper lobe	726	32.2(28.5-36.5)	<0.001	344	89	19.4(14.0-25.0)	0.668	34
	Right middle lobe	152	31.0(24.6-48.0)		59	26	19.0(16.2-48.7)		8
	Right lower lobe	435	31.2(25.9-39.7)		180	53	20.0(14.2-30.9)		30
	Left upper lobe	699	27.2(24.9-31.7)		294	104	22.0(17.8-29.3)		49
	Left lower lobe	426	29.0(26.2-35.8)		166	57	16.9(13.2-29.0)		23
	Others^a^	516	18.9(16.2-20.6)		223	121	19.0(16.0-25.2)		49
Central location	Central	517	23.8(21.6-25.2)	<0.001	225	88	19.0(16.2-22)	0.573	46
	Peripheral	1431	34.0(30.6-39.0)		593	308	25.6(21.8-49)		130
	Unknown^b^	1006	32.2(26.6-38.6)		448	54	19.4(15.7-27)		17
Pathological types	Adenocarcinoma	938	25.4(23.4-27.9)	0.002	392	—	—	—	—
	Squamous carcinom	1683	27.7(26.3-30.6)		722	—	—	—	—
	Adenosquamous carcinoma	120	45.2(37.9-58.9)		55	—	—	—	—
	Other	213	23.2(18.1-28.5)		97	—	—	—	—
Differentiation	High	181	51.3(36.2-66.8)	<0.001	81	—	—	—	—
	Moderate	866	38.1(35.4-43.4)		355	—	—	—	—
	Low	663	21.9(19.3-24.0)		292	—	—	—	—
	Undifferentiated	558	19.2(17.3-21.3)		257	—	—	—	—
Diagnostic method	Biopsy	1476	20.7(19.3-22.3)		614	387	20.0(17.8-23.0)	0.054	171
	Surgery	1085	60.4(53.9-67.5)	<0.001	451	23	34.0(21.8-NA)		8
	Cytology	393	14.6(12.6-16.6)		201	40	12.4(7.6-34.1)		14
Therapeutic regimen	Simple chemotherapy	1131	18.3(16.9-19.5)		490	238	17.4(15.0-22.0)		104
	Surgery/Radiotherapy/Chemotherapy	137	43.9(38.6-54.0)	<0.001	49	—	—	—	
	Surgery/ Radiotherapy	87	32.3(23.5-42.1)		42	—	—	—	
	Surgery/ Chemotherapy	434	79.6(68.3-NA)		203	—	—	—	
	Radiotherapy / Chemotherapy	449	22.3(20.4-25.7)		190	171	21.0(18.0-25.7)	0.051	72
	Simple surgery	488	57.0(51.0-75.6)		175	—	—	—	
	Simple radiotherapy	197	18.0(14.4-24.3)		105	16	18.4(6.97-NA)		7
	Others	31	14.9(8.93-21.2)		12	25	34.0(25.23-NA)		10

NSCLC, non-small-cell lung cancer; SCLC, small-cell lung cancer; OS, overall survival.

a: Tumors locating at two or more lobes were named “other”.

b: Tumors which could not be distinguished as central or peripheral were named “unknown”.

For patients with SCLC, most therapies involved C (n=342), RC (n=243), and R (n=23). The other therapies were combined as “others,” which included SRC (n=12), SC (n=12), S (n=9), SR (n=0) and others (n=2).

### Independent prognostic factors in the training set of NSCLC and SCLC patients

The univariate analysis results of NSCLC and SCLC patients in the training set are listed in Table 1.

Among all occupations in the NSCLC training dataset, farmers had the lowest survival rates, followed by public sector employees, freelance or self-employed individuals, enterprise or company employee/worker, and others (P=0.003). Urban residents with basic medical insurance (URB) had higher survival rates, compared to urban employees with basic medical insurance (UEB), new rural cooperative medical scheme (NRC) workers, self-pay (Self) patients, or patients on other health insurance plans (P<0.001). Clinical stage T (P<0.001), clinical stage N (P<0.001), clinical stage M (P<0.001), and differentiation phase (P<0.001) also affected survival rates. Patients with tumors in the right upper lobe of the lung had the most favorable survival rates, followed by the right lower lobe, right middle lobe, left lower lobe, left upper lobe and other locations (P<0.001). Patients with centrally located tumors had poorer survival rates, compared to patients with peripheral tumors (P<0.001). Patients with adenosquamous carcinoma had better survival rates than patients with squamous carcinoma and adenocarcinoma (P=0.002). Patients who accepted the proposed diagnostic method had higher survival rates. For example, surgery was superior to biopsy and cytology methods (P<0.001). Patients with lung cancer usually agreed to the proposed regimen, which included—individually or in combination—surgery, chemotherapy, or radiotherapy. With regard to survival rates, SC was the most successful treatment, followed by S, SRC, SR, RC, C, R, and others (P<0.001). No significant survival differences were found among gender groups (men vs. women; P=0.672), age groups(<60 vs. 60-70 vs. >70 years; P=0.095) and smoking habit groups (<30 vs. 30-40 vs. >40 pack-y; p=0.727)

In the NSCLC group, higher education levels were associated with a better prognosis (P<0.001). In the SCLC group, patients who attended junior and senior high school had better survival rates than patients who attended primary school or below or patients who were undergraduates or over (P=0.002). Health insurance plan also impacted survival (NRC > URB > Self > UEB > Other; P=0.002), whereas other factors were not correlated with survival (Table 1).

A backward (i.e., step-down) Cox regression analysis was used to model the prognostic predictive value of several parameters from the NSCLC training cohort, which were age, occupation, health insurance plan, clinical T stage, N stage, and M stage, central location, differentiation phase, diagnostic method, and therapeutic regimen. In the SCLC group, the model included gender, health insurance plan, clinical stage, and therapeutic regimen (Table 2).

**Table 2:** Cox proportional hazards regression analysis

		NSCLC			SCLC		
Variable		Hazard ratio	95%CI	*P*	Hazard ratio	95%CI	*P*
Gender	Female	—	—	—	Reference		
	Male	—	—	—	1.27	0.97-1.57	0.116
Age	<60	Reference			—	—	—
	60-70	1.05	0.95-1.15	0.371	—	—	—
	>70	1.25	1.07-1.43	0.016	—	—	—
Occupation	Enterprise or company employee/worker	Reference			—	—	—
	Famer	1.16	1.02-1.30	0.040			
	Public sector employee	1.12	1.00-1.24	0.057	—	—	—
	Freelance or self-employed	0.91	0.69-1.13	0.384	—	—	—
	Others	0.91	0.68-1.13	0.384	—	—	—
Health insurance	New rural cooperative medical scheme	Reference			Reference		
	Other	1.42	1.25-1.60	<0.0001	2.17	1.81-2.52	<0.001
	Self pay	1.27	1.13-1.41	0.001	1.65	1.33-1.96	0.002
	Urban employees basic medical insurance	1.09	0.91-1.26	0.357	1.42	1.12-1.73	0.023
	Urban residents basic medical insurance	1.01	0.78-1.23	0.958	1.22	0.78-1.67	0.371
Clinical T stage		1.10	1.05-1.15	<0.0001	—	—	—
Clinical N stage		1.16	1.12-1.20	<0.0001	—	—	—
Clinical M stage		1.34	1.24-1.43	<0.0001	—	—	—
Clinical stage	—/ Extensive	—	—	—	Reference		
	—/ Limited	—	—	—	0.82	0.60-1.03	0.065
Central location	Central	Reference			—	—	—
	Peripheral	0.91	0.81-1.02	0.068	—	—	—
	Unknown^a^	0.88	0.75-1.00	0.041	—	—	—
Differentiation		1.05	0.99-1.10	0.096	—	—	—
Diagnostic method	Biopsy	Reference					
	Surgery	0.74	0.49-0.99	0.018	—	—	—
	Cytology	1.22	1.09-1.35	0.002	—	—	—
Therapeutic regimen	Simple chemotherapy	Reference			Reference		
	Surgery/Radiotherapy/ Chemotherapy	0.97	0.66-1.27	0.819	—	—	—
	Surgery/ Radiotherapy	1.09	0.74-1.43	0.631	—	—	—
	Surgery/ Chemotherapy	0.65	0.37-0.93	0.002	—	—	—
	Radiotherapy / Chemotherapy	0.91	0.79-1.04	0.149	0.76	0.53-0.99	0.021
	Simple surgery	0.82	0.54-1.11	0.179	—	—	—
	Simple radiotherapy	0.93	0.76-1.11	0.453	0.79	0.21-1.37	0.424
	Others	1.23	0.86-1.60	0.279	0.58	0.07-1.09	0.034

NSCLC, non-small-cell lung cancer; SCLC, small-cell lung cancer; CI, confidence interval.

a: Tumors which could not be distinguished as central or peripheral were named “unknown”.

### Prognostic nomogram for survival

**Figure 1:**
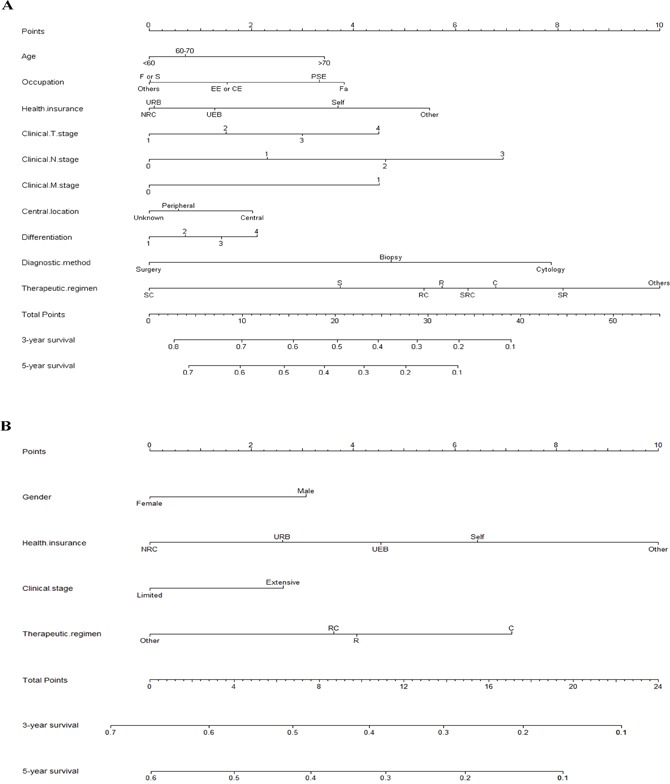
Prognostic nomograms for patients with **(A)** non-small-cell lung cancer and **(B)** small-cell lung cancer. CE, company employee; EE, enterprise worker; F, freelance; Fa, farmer; NRC, new rural cooperative medical scheme; PSE, public sector employee; F or S, freelance or self-employed; Self, self-pay; UEB, urban employees basic medical insurance; URB, urban residents basic medical insurance. Treatment regimens: C, simple chemotherapy; R, simple radiotherapy; RC, chemotherapy/radiotherapy; S, simple surgery, SC, surgery/chemotherapy; SR, surgery/radiotherapy; SRC, surgery/chemotherapy/radiotherapy. Differentiation: 1, High; 2, Moderate; 3, Low; 4, Unkown.

A nomogram that incorporated selected prognostic factors was established (Figure 1, Supplementary Table 1). The plot of the NSCLC patients shows that the therapeutic regimen and diagnostic method parameters accounted for the largest contribution to OS prognosis. For the survival rate, the contribution of the clinical N stage, health insurance plan, clinical T stage, clinical M stage, occupation, age, central location, and differentiation phase parameters were significantly lower. The nomogram for SCLC with patients showed that health insurance plan and therapeutic regimen parameters provided the greatest contribution, followed by gender and clinical stage parameters. Each variable was assigned a score on the point scale, which made it possible to draw a straight line down to the survival line from the total point scale by adding up the score of each variable to get the estimated probability of survival at each time point.

### Calibration and validation of the nomogram

**Figure 2:**
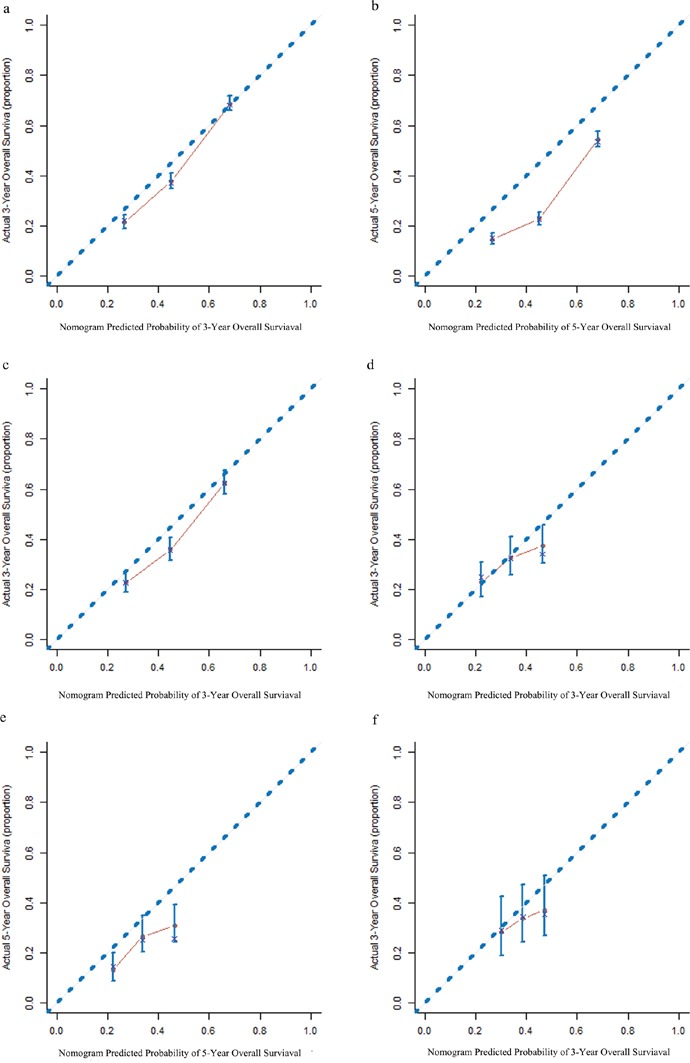
The calibration curves for predicting survival of patients with non-small-cell lung cancer at **(a)** 3 years and **(b)** 5 years in the primary cohort, and at **(c)** 3 years in the validation cohort; and for predicting survival of patients with small-cell lung cancer at **(d)** 3 years and **(e)** at 5 years in the primary cohort, and at **(f)** 3 years s in the validation cohort. The nomogram-predicted probability of the overall survival (OS) is plotted on the *x*-axis. The actual OS is plotted on the *y*-axis.

The calibration data for the 3-year OS plots had optimal agreement between the nomogram prediction and actual observation, whereas the 5-year OS data had acceptable agreement for the NSCLC and SCLC cohort training datasets. Calibration of the validation dataset showed nearly the same results as the training dataset (Figure 2). The C-index of our nomogram for predicting OS was higher in the primary cohort (0.67; 95%CI, 0.65-0.69), compared to the C-index values obtained from the TNM staging system in the NSCLC cohort (0.64; 95%CI, 0.62-0.66; *P* = 0.01). The C-index of our nomogram (0.60; 95%CI, 0.55-0.65) was superior to clinical stage, limited / extensive stage, and diagnosis (0.53; 95%CI, 0.47-0.59) for the SCLC cohort, although this was not significantly different (*P* =0.12).

Using the NSCLC validation cohort, the C-index of our nomogram (0.65; 95%CI, 0.61-0.69) was also higher than that of TNM staging diagnosis (0.63; 95%CI, 0.59-0.67; *P* = 0.39). Furthermore, the C-index of our nomogram (0.60; 95%CI, 0.54-0.66) was greater than that of the clinical stage diagnosis for the SCLC group (0.52; 95%CI, 0.44-0.60; *P* = 0.25); however, no significant difference existed between the nomograms.

### Performance of the nomogram in stratifying patient risk

**Figure 3:**
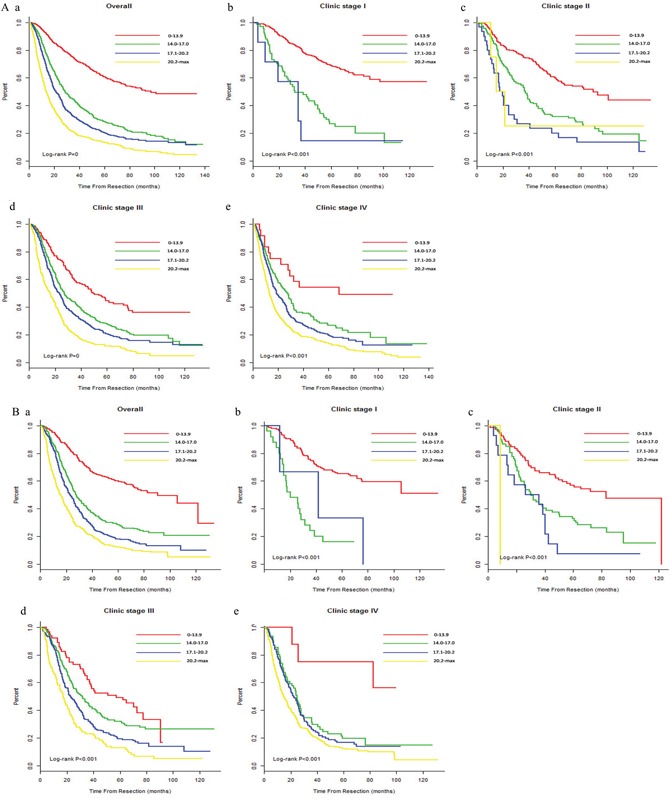
Risk group stratification within each TNM stage **(A)** in the primary cohort (a, all patients; b-e, stages) and **(B)** in the validation cohort of the NSCLC patients (a, all patients; b-e, stages) NSCLC, non-small-cell lung cancer; SCLC, small-cell lung cancer; TNM, tumor-node-metastasis.

Three cutoff values for the NSCLC training cohort were determined by grouping patients into four subgroups, after sorting their respective total scores (score: 0–13.9, 14.0-17.0, 17.1-20.2, and ≥20.2). Each subgroup showed a distinct prognosis between the Kaplan–Meier curves within four clinical stages(*P*<0.001 for all; Supplementary Table 1 and Figure 3A). When these cutoff values were applied to the validation cohort dataset, the plots also represented a significant distinction beyond the TNM categories (*P* < 0.001 for all, Figure 3B).

**Figure 4:**
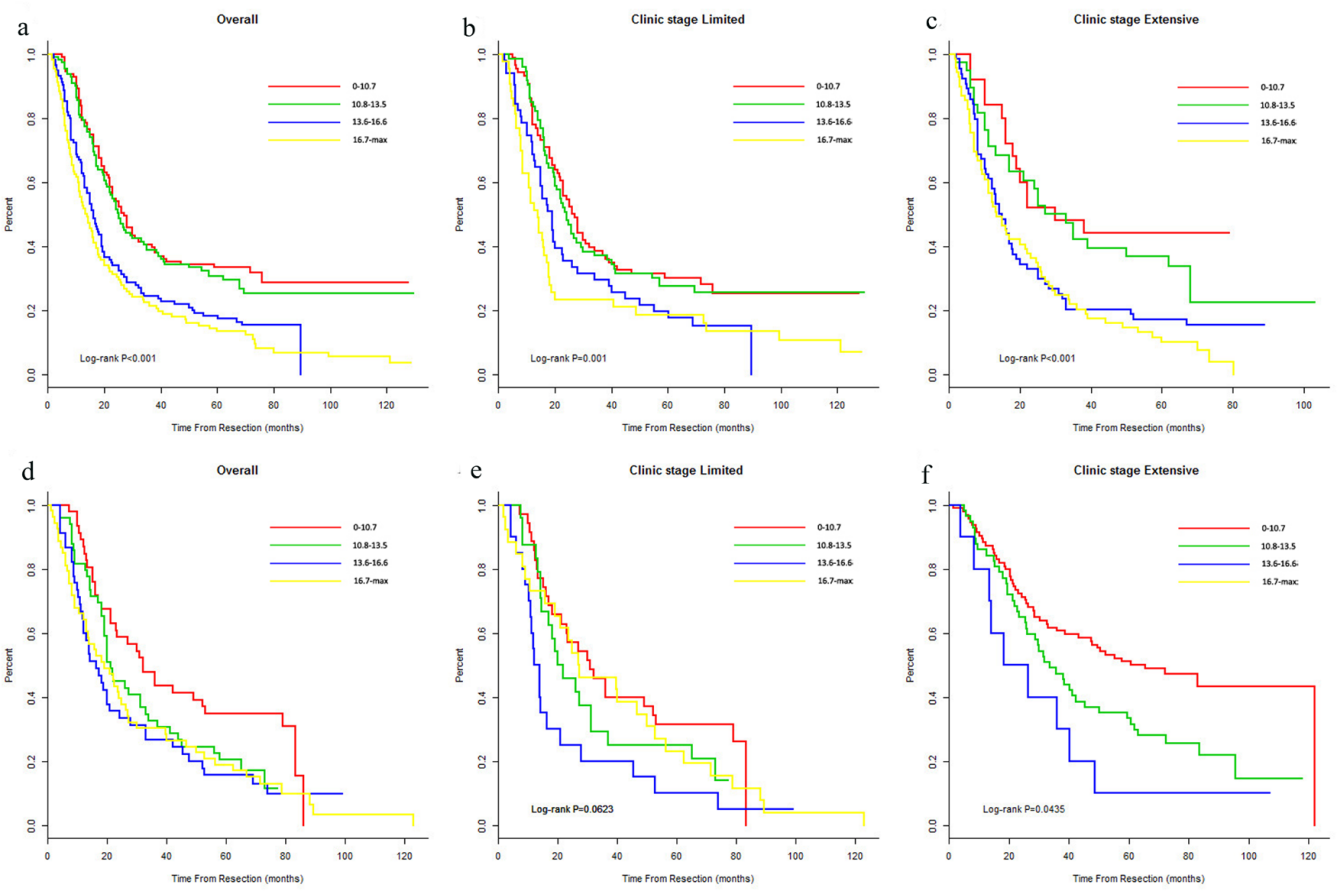
The risk group stratification within each clinical stage in the primary cohort (**a**, all patients; **b-c**, stages) and in the validation cohort of the SCLC patents (**d**, all patients; **e-f**, stages) NSCLC, non-small-cell lung cancer; SCLC, small-cell lung cancer.

We also grouped the SCLC dataset into four subgroups in the training and validation cohorts (score: 0–10.7, 10.8-13.5, 13.6-16.6, and ≥16.7). From the plot, the Kaplan–Meijer curves in the training cohort demonstrated significant distinction prognosis beyond the limited stage (*P*=0.001) and the extensive stage (*P*<0.001). In the validation cohort, only the extensive stage curves showed a significant difference between the four subgroups (*P*=0.0435, Figure 4).

## DISCUSSION

Nomograms reliably quantify risk by incorporating and illustrating important factors for oncologic prognoses. In several types of cancers, nomograms generate a more precise prediction, compared to traditional TNM staging systems. The aim of the current study these studies was to build on previous efforts to develop prognostic nomograms for predicting survival rates in patients with non-small cell and small cell lung cancers. The goal was to identify additional prognostic parameters that could be used to predict optimal treatments, disease trajectory, and OS. We specifically focused on identifying parameters that could improve the capability of nomograms to predict OS in these patients.

The entire cohort was from a single tertiary cancer hospital with advanced medical care facilities. The parameters of the patients with NSCLC included age, occupation, health insurance plan, clinical TNM stage, central location, differentiation phase, diagnostic method, and therapeutic regimen—all of which were identified as prognostic factors. Among these parameters, age, clinical TNM stage, central location, and differentiation phase had high concordance with previous studies [4, 15, 16]. However, occupation, health insurance plan, diagnostic method, and therapeutic regimen parameters were not included in previous NSCLC prognostic survival nomograms. The occupation parameter revealed that farmers had the lowest survival, which was probably associated with their low income, as lung cancer treatment costs can be high in China. The health insurance plan parameter also revealed correlations. This study showed that the survival rates of patients with standard health insurance plans were superior to those with self-finance (i.e., Self) or other types of health insurance. We believe that standard health insurance plans increase survival by reducing the economic burden of healthcare because they provide more advanced treatments at no extra cost. Health authorities should consider this factor when instituting health insurance plan policies. The therapeutic regimen parameter showed a clear correlation with OS. The SC and S regimens positively impacted survival, which concurs with previous studies demonstrating that administering adjuvant chemotherapy in stage II and III cancers was beneficial [20, 21]. The diagnostic method parameter revealed that surgery significantly improved survival, compared to biopsy and cytology. Biopsy is the preferred method to diagnose cancer. However, patients sometimes could not be diagnosed by obtaining available pathological tissues through bronchoscopy because of poor location or visualization of advanced tumor. The physician would then perform surgery or arrange a cytology examination for a diagnosis. On the other hand, cytology is commonly available for patients with advanced lung cancer who are unsuitable for surgery. So probably the diagnostic method way predicts survival rate via correlating therapeutic regimen.

In the SCLC cohort, the nomogram model included the parameters of gender health insurance plan, clinical stage, and therapeutic regimen. Female and clinical limited stage parameters were positively associated with survival. The health insurance plan parameter paralleled the results from the NSCLC cohort, which indicated that this parameter identified financial status rather than the specific disease *per se*. The therapeutic regimen parameter affected survival. The “other” category focused on surgery, which included SRC, SC, and S. This category showed increased survival, which outperformed the RC, R, and C treatments. These results differed significantly from those of Xie et al. [6], and indicated that additional factors are involved. These differences often provide critical insights for refining treatments. Further analysis of these cohorts and datasets will be required to resolve this apparent anomaly.

Nomogram validation is essential to prevent a model from potentially overestimating the predictive performance of the present data and to determine the generalizability of the nomogram to patients [22]. In present study, the calibration plots showed “optimal” agreement for the 3-year OS and “acceptable” agreement for the 5-year OS between the predicted and actual observed values for the NSCLC and SCLC training dataset cohorts. Thus, the predictive performance was more repeatable and reliable in the 3-year OS rate than in the 5-year OS rate. In present study, the follow-up time of most patients with NSCLC and SCLC were longer than 5 years (median follow-up time: 6.5 years and 6.3 years, respectively). In this long period, the survival status of most patients may have been affected by intervening measures such as psychological factors, behavioral factors, and by other therapeutic methods that we could not follow. Therefore, the model we established, based on demographic and clinicopathologic characteristics parameters, was more suitable for predicting recent survival: i.e. it was more precise for predicting the 3-year survival rate than the 5-year. Furthermore, the NSCLC and SCLC models fit the validation groups. Our nomogram of NSCLC patients outperformed the TNM staging system for predicting OS in patients with LC evaluated using the C-index (0.67 *v* 0.64, *P* =0.01). However, in the validated cohort, the difference in the C-index between the nomogram and TNM staging system (0.65 v 0.63, *P*=0.39) was not significant. In the validation group, compared to the clinical III and IV stages, the clinical I and II stages only accounted for 26.5% of the whole sample. This big inequality may have caused prediction bias in the model. Therefore, a larger validatiton sample is required.

The cohorts were further stratified into four risk subgroups within their respective TNM staging categories based on quartile deviation in training set. These plots of NSCLC showed distinctive survival. For the SCLC cohort, the predictive accuracy of the nomogram for OS was far superior to the clinical TNM staging system for the primary dataset (limited vs. extensive: 0.60 vs. 0.53) and the validation dataset (limited vs. extensive: 0.60 vs. 0.52). However, the *P* values indicated no significant differences in the primary dataset (*P* = 0.12) or the validation dataset (*P* = 0.25). The four risk groups in the limited and extensive stages exhibited significant prognostic capabilities for the primary and validation datasets, except for the limited stage in the validation cohort, which had no significant difference (*P*=0.624). These three insignificant points mentioned values (i.e., *P* value was not 0.05) may be because of the small sample size. Future studies are needed to validate these findings.

A nomogram based on the novel combination of the prognostic parameters of occupation, health insurance plan, diagnostic method and therapeutic regimen was constructed to predict NSCLC and SCLC survival. To the best of our knowledge, this is the first time NSCLC and SCLC survival nomograms have been constructed using this combination of parameters. The study used a cohort that was sufficiently large to allow statistical analysis and included long-term patient follow-up to improve data accuracy. Among the prognostic parameters used in this study, therapeutic regimen was the most significant prognostic factor for predicting NSCLC and SCLC lung cancer OS. It contributed the most to the OS, and is a very crucial variable to clinical physicians and patients, both of whom can easily and accurately use this scoring system to determine the optimal therapeutic regime preliminarily. It has never been identified in previous related studies. Using this tool, physicians can divide patients into different risk groups and care for them accordingly. Our nomogram constructs, more importantly, provide more accurate prognostic models than the TNM staging system. Currently, standardized therapy regimens probably causing over-treatment or under-treatment are applied generally used by oncologists, which influence the life quality and survival time to a large extent, because they do not involve in tumor and individual heterogeneity. However, the nomogram used to predict survival considers some individual factor such as demographic, clinical and serum parameters. In future studies, more advanced parameters like molecular factors may be found to predict survival, and then individual and targeted therapy could be deeply applied for patients with lung cancer [23].

Our study also has some limitations. First, the prognostic parameters used in the construction of our nomograms involved typical routine clinical data. More advanced clinical parameters that are prognostic of survival such as tumor size [24], FEV1 [25], lymphatic permeation [26], lymphovascular invasion [27], and molecular factors [23] were not included in the design of our nomogram because there were too few incomplete clinical data. An advantage of using basic low-cost clinical parameters is that the data will be widely available, which simplifies performing multicenter studies. The simplest solution is always the best solution. A second possible limitation is that our nomograms were constructed, based on clinical data from a single institute. However, it is becoming increasingly common to analyze multiple retrospective studies to increase the size of a dataset and to reduce center-specific effects. Single-center studies provide an excellent starting point; if current trends continue, then the clinical data on which this article are based will be used in in future studies. An additional point is that the dataset for the SCLC nomogram was much smaller than the NSCLC dataset and needs further validation using a larger cohort. A third limitation was that to prevent the overfitting of our multivariate Cox model, the number of variables was limited. It is possible that the association was confounded or mediated by another variable that we did not consider. Our findings need to be confirmed using multiple models and larger datasets from multiple clinics.

## PATIENTS AND METHODS

### Patient population

The patient population in this retrospective study comprised patients with NSCLC (n=5384) and SCLC (n=647) diagnosed from January 2000 to December 2009. The clinical data were collected from the tertiary cancer hospital affiliated with the Xiang Ya Medical School of Central South University in Changsha, China. This study ended on December 31, 2014. Ethical approval was acquired from the institutional review board at the hospital.

For patients with NSCLC and SCLC, inclusion criteria included histopathological examination, acceptance of all main treatments (surgery, chemotherapy, radiotherapy and other therapy like Chinese traditional therapy, biological therapy, etc.) in the hospital, and no history of other malignant tumors or previous anticancer therapy. The exclusion criteria were uncertain tumor origin, probable metastatic lung cancer, and mixed histopathological primary lung cancer. In the data screening process, variables with more than 10% missing values were not included in study analysis. Moreover, data records on eligible variables with any missing value were omitted from the data analysis. In total, 4220 NSCLC patients and 643 SCLC patients were enrolled in the study group. To test the generalizability of the model, we randomly divided the entire database into a training dataset (NSCLC, n=2954; SCLC, n=450) and a validation dataset (NSCLC, n=1266; SCLC, n=193) at a ratio of 7:3. Each patient signed an informed consent document in this study.

### Data collection

The data collection form covered four areas: (1) the demographic characteristics (i.e., age, gender smoking history [in pack-years], occupation, education, and health insurance plan); (2) the clinical information (i.e., date of diagnosis, pathological type, clinical stage, clinical TNM stage, tumor location, central location, and differentiation phase); (3) the therapeutic regimen (i.e., surgery/radiotherapy/chemotherapy [SRC], surgery/radiotherapy [SR], surgery/chemotherapy [SC], simple surgery [S], simple chemotherapy [C], and simple radiotherapy [R]); and (4) the follow-up data (i.e., follow-up date, outcome, outcome date, source of acquired outcome, and survival time). The patients’ follow-up data were obtained by reviewing their medical files. Any relevant missing data were acquired by contacting the patient directly. Patient survival times calculated from the first date of pathological diagnosis data were registered until the patient's death or until the last registered contact. Clinical stage was determined via pathological diagnosis using the World Health Organization classification system, which is based on the seventh edition of the American Joint Committee on cancer TNM staging system [28].

Two alternatives were possible regarding central location: central or peripheral. A tumor was classified as centrally located if its center was in the medial third of the lung parenchyma, and as lateral if two thirds of the locations were classified as peripheral tumors.

The therapeutic regimen of the patient was normally determined by the responsible physician using the patient's diagnosis data, based on the latest version by the National Comprehensive Cancer Network (NCCN). For some special and complicated cases, the therapeutic regimen was decided in a consultation meeting with a senior physician in the department. It was then administered within a month. The surgery types comprised of wedge/segmentectomy, lobectomy, bi-lobectomy, and pneumonectomy, which included video-assisted thoracoscopic surgery and thoracotomy. Chemotherapy comprised of platinum-based doublets such as cisplatinum/paclitaxel and cisplatinum/pemetrexed. Patients who received radiotherapy were administered a dose ranging 45–64.8 Gy (i.e., 1.8-2.0 Gy/day) with 6 MV X-ray. Moreover, radiotherapy involved conventional radiotherapy, three dimensional conformal radiotherapy, and intensity-modulated radiotherapy. All data used for this study were managed by an authorized biostatistician and were collected by qualified medical personnel.

### Statistical analysis

#### Construction of the nomogram

In the training group, survival curves for all variables were generated using Kaplan–Meier estimates and were statistically compared using the log-rank test. Based univariate analysis, variables with values of *P<*0.1 underwent Cox regression analysis to create a prediction model with a backward step-down process performed using the Akaike information criterion stopping rule [29].

#### Validation and calibration of the nomogram

Internal validation of the training dataset was performed using 1000 bootstrap resamples and the nomogram was applied to the validation cohort for external validation. The performance of the model to prognostically predict outcomes was evaluated using the concordance index (C-index) which ranged from 0.5 (random chance) to 1.0 (perfect discrimination). The nomogram for the 3- and 5-year OS was calibrated by comparing the predicted survival rates with the observed survival rates.

#### Risk group stratification, based on nomogram analysis

The sum-score for each patient was calculated, based on the established model. Patients were then divided into four risk groups beyond clinical stage. Cutoff values were determined using their sum scores from the training dataset (from highest to lowest). The Kaplan–Meier survival curves were delineated, based on four different groups with log-rank comparison. All data management and statistical analyses were performed using SPSS (version 19.0, SPSS, Chicago, IL, USA), and R software (version 3.3.0; R Foundation for Statistical Computing, Vienna, Austria) with the survival, and RMS package (Regression Modeling Strategies, Inc., Newark, CA). All statistical tests were two-sided, and a p-value <0.05 was statistically significant.

## CONCLUSIONS

The NSCLC and SCLC nomograms for predicting the survival of lung cancer patients were established and validated in an objective and precise manner. Our results showed that the treatment regimen parameter is predictive of survival in lung cancer patients. This information makes it possible for clinicians and patients to choose a treatment regimen that is based on patient-specific parameters rather than on average predefined treatment regimens. The nomograms will provide clinicians and patients with critical information needed to make informed treatment decisions. Furthermore, the clinical data collected during the course of this study could be used in future literature review-based studies.

## SUPPLEMENTARY MATERIALS TABLE

Supplementary Table 1 Prognostic score according to nomogram plots
