# Supplementary material for: Development and validation of two prognostic nomograms for predicting survival in patients with non-small cell and small cell lung cancer
Source: Oncotarget. 2017 Aug 2;8(38):64303–16. doi: 10.18632/oncotarget.19791 (PMC5610004; doi:10.18632/oncotarget.19791)
Supplement: Supplementary Table 1 [file oncotarget-08-64303-s001.doc]

**Supplementary Table 1: Prognostic score according to nomogram plots**

|  |  | **NSCLC** | | | **SCLC** | |
| --- | --- | --- | --- | --- | --- | --- |
| **Variable** |  | | **Prognostic score** | **Estimated 5-year overall** | **Prognostic score** | **Estimated 5-year overall** |
| Gender | Male | | — | — | 3.1 | — |
|  | Female | | — | — | 0 | — |
| Age,years | <60 | | 0 | — |  | — |
|  | 60-70 | | 0.7 | — |  | — |
|  | >70 | | 3.4 | — |  | — |
| Occupation | Enterprise or company employee/worker | | 1.5 | — | — | — |
|  | Famer | | 3.8 | — | — | — |
|  | Public sector employee | | 3.3 | — | — | — |
|  | Freelance or self-employed | | 0 | — | — | — |
|  | Others | | 0 | — | — | — |
| Health insurance | New rural cooperative medical scheme | | 0 | — | 0 | — |
|  | Urban residents basic medical insurance | | 0.1 | — | 2.6 | — |
|  | Urban employees basic medical insurance | | 1.3 | — | 4.6 | — |
|  | Self pay | | 3.7 | — | 6.4 | — |
|  | Other | | 5.5 | — | 10.0 | — |
| T stage | T1 | | 0 | — | — | — |
|  | T2 | | 1.5 | — | — | — |
|  | T3 | | 3.0 | — | — | — |
|  | T4 | | 4.5 | — | — | — |
| N stage | N0 | | 0 | — | — | — |
|  | N1 | | 2.3 | — | — | — |
|  | N2 | | 4.6 | — | — | — |
|  | N3 | | 6.9 | — | — | — |
| M stage | M0 | | 0 |  |  |  |
|  | M1 | | 4.5 |  |  |  |
| Clinic stage | — / Limited | | — | — | 0 | — |
|  | — / Extensive | | — | — | 2.6 | — |
| Central location | Central | | 2 | — | — | — |
|  | Peripheral | | 0.6 | — | — | — |
|  | Unknown^a^ | | 0 | — | — | — |
| Differentiation | High | | 0 | — | — | — |
|  | Moderate | | 0.8 | — | — | — |
|  | low | | 1.5 | — | — | — |
|  | Undifferentiated | | 2.1 | — | — | — |
| Diagnostic method | [Biopsy](javascript:void(0);) | | 4.8 | — | — | — |
|  | Surgery | | 0 | — | — | — |
|  | [Cytology](javascript:void(0);) | | 7.9 | — | — | — |
| Therapeutic regimen | Simple chemotherapy | | 5.8 | — | 7.1 | — |
|  | Surgery/Radiotherapy/ Chemotherapy | | 6.3 | — | — | — |
|  | Surgery/ Radiotherapy | | 8.1 | — | — | — |
|  | Surgery/ Chemotherapy | | 0 | — | — | — |
|  | Radiotherapy/ Chemotherapy | | 4.4 | — | 3.6 | — |
|  | Simple surgery | | 3.8 | — |  | — |
|  | Simple radiotherapy | | 4.6 | — | 4.1 | — |
|  | Others | | 10.0 | — | 0 | — |
| Total prognostic score | 0-13.9 / 0-10.7 | | — | 59.0 | — | 33.0 |
|  | 14.0-17.0 / 10.8-13.5 | | — | 29.0 | — | 31.0 |
|  | 17.1-20.2 / 13.6-16.6 | | — | 21.0 | — | 18.0 |
|  | 20.2-max / 16.7-max | | — | 12.0 | — | 14.0 |

Notes: Abbreviation: NSCLC, non-small-cell lung cancer; SCLC, small-cell lung cancer.

a: Tumors which could not be distinguished as central or peripheral were named “unknown”.
